# Integrative Analysis of Lipid Profiles in Plasma Allows Cardiometabolic Risk Factor Clustering in Children with Metabolically Unhealthy Obesity

**DOI:** 10.1155/2020/2935278

**Published:** 2020-08-11

**Authors:** Elena Cristina Castillo, Leticia Elizondo-Montemayor, Carmen Hernández-Brenes, Dariana G. Rodríguez-Sánchez, Christian Silva-Platas, Luis Martín Marín-Obispo, Nora A. Rodríguez-Gutierrez, Víctor Treviño, Gerardo García-Rivas

**Affiliations:** ^1^Tecnologico de Monterrey, Escuela de Medicina y Ciencias de la Salud, Cátedra de Cardiología y Medicina Vascular. Monterrey, Nuevo León, Mexico; ^2^Tecnologico de Monterrey, Escuela de Medicina y Ciencias de la Salud, Centro de Investigación Clínica en Nutrición y Obesidad. Monterrey, Nuevo León, Mexico; ^3^Tecnologico de Monterrey, Escuela de Ingeniería y Ciencias. Monterrey, Nuevo León, Mexico; ^4^Tecnologico de Monterrey, Centro de Biotecnologia-FEMSA. Monterrey, Nuevo León, Mexico; ^5^Hospital Regional de Alta Especialidad Materno Infantil, Guadalupe, Nuevo León, Mexico; ^6^Tecnologico de Monterrey, Escuela de Medicina y Ciencias de la Salud, Cátedra de Bioinformática. Monterrey, Nuevo León, Mexico; ^7^Tecnologico de Monterrey, Hospital Zambrano Hellion, TecSalud, Centro de Investigación Biomédica. San Pedro Garza García, Nuevo León, Mexico; ^8^Tecnologico de Monterrey, Hospital Zambrano Hellion, TecSalud, Centro de Medicina Funcional. San Pedro Garza García, Nuevo León, Mexico

## Abstract

Hypertension, central obesity, hyperglycemia, and dyslipidemia are key risk factors for cardiovascular disease. However, the specific factors contributing to the development of unfavorable cardiometabolic characteristics in children with obesity are unknown. In this study, we investigated the cross-sectional relationships between cytokines, irisin, and fatty acid (FA) composition in plasma in school-age children with metabolically healthy and unhealthy obesity (MHO and MUO, respectively) of the same age and body mass index and waist circumference percentiles. We compared the data with that of children with normal weight (NW). We found that inflammatory cytokines and low irisin plasma concentrations are associated with obesity but not with cardiometabolic risk (CMR). Lipid profiles showed that children with MUO have a distinctive FA profile compared with children with MHO and NW, whereas children with MHO shared 88% of the FA profile with the NW group. Among all FAs, concentration of myristic acid (14 : 0), arachidic acid (20 : 0), and n-3 polyunsaturated FAs (PUFAs) was higher in children with MHO, whereas n-6 PUFAs such as arachidonic acid (20 : 4n6) had a significant contribution in defining MUO. These data suggest that the plasma FA profile is not only a central link to obesity but also may act as an indicator of CMR presence.

## 1. Introduction

Given the lack of consensus on obesity-associated risk factors in the pediatric definition of metabolic syndrome, the American Academy of Pediatrics recommends focusing on cardiovascular risk factor clustering (blood pressure, central obesity, hyperglycemia, high triglycerides (TG), and decreased high-density lipoprotein cholesterol [HDL-c]) to identify the risk for cardiovascular disease (CVD) [1]. In this context, unraveling the factors that contribute to obesity (OB) and the development of cardiometabolic risk (CMR) has been a challenge. It has been suggested that the emerge of CMR factors depends on age and waist circumferences (WC) [2, 3]. However, children with OB who are resilient to unfavorable CMR, known as metabolically healthy obesity (MHO), has been described [4]. Data suggest that other factors are associated with the emergence of CMR, leading to metabolically unhealthy obesity (MUO). Of note, fatty acids (FAs), inflammatory markers, and adipomyokines are key influencing molecules that might contribute to a metabolic transition from MHO to MUO. For instance, diet influences inflammation and irisin (adipomyokine) concentrations that contribute to the development of insulin resistance and type 2 diabetes (T2D) [5, 6]. Particularly, saturated fatty acids (SFAs) have proinflammatory effects, whereas n-3 PUFAs, such as eicosapentaenoic acid (EPA, 20 : 5n3) and docosahexaenoic acid (DHA, 22 : 6n3), counteract these effects by reducing proinflammatory cytokines secretion by macrophages and by diminishing insulin resistance [7]. Moreover, patients with a higher level of systemic inflammation have a higher risk of developing T2D and CVD, even in nonobese patients [8]. Irisin, which is a thermogenic adipomyokine with anti-inflammatory properties [9], induces insulin synthesis [10] to improve glucose homeostasis [11] and is negatively associated with T2D in children [12, 13]. Therefore, our goal was to determine whether FAs, cytokines, and irisin concentrations are differentially associated with MHO and MUO. Our study was performed in children with OB subdivided into MHO and MUO blocking age and anthropometric factors compared with children with normal weight (NW). Our study provides an insight into the contribution of FAs for defining cardiometabolic risk clustering in children with MHO and MUO and might be helpful in determining awareness for the medical care beyond weight loss in children with obesity.

## 2. Materials and Methods

### 2.1. Population

Mexican children from Monterrey [14] aged 6 to 12 years were classified according to their BMI percentile as children with NW (>5^th^ and <85^th^ percentiles) or OB (≥95^th^ percentile) [15] and in MHO or MUO based on the number of CMR factors [1, 3, 16, 17]: blood pressure (systolic or diastolic ≥90^th^ percentile for age, gender, and height), hyperglycemia (≥100 mg/dL), hypertriglyceridemia (≥110 mg/dL), and low HDL-c (≤40 mg/dL). MHO was defined as children with none or one CMR factor and MUO as children with two or more factors as defined by other authors [16, 17]. The total population was selected as previously established [14]. This cross-sectional study includes samples of 34 children (18 girls and 16 boys) with NW and 80 children with OB (40 girls and 40 boys) was selected. The OB group was further divided into two groups: MHO (36 children) and MUO (44children). Diet and physical activity were not controlled during the study. This research was carried out in accordance with the ethical principles of the Declaration of Helsinki. Approvals were obtained from the Ethics and Research Committees of the School of Medicine Tecnologico de Monterrey and the government state education authorities of Nuevo Leon, México. All parents or legal guardians gave their written informed consent.

### 2.2. Anthropometric Measurements

All the anthropometric measures were determined, as previously described [14]. Briefly, body weight and height were measured to obtain the BMI (kg/m^2^) and the percentile based on World Health Organization (WHO) tables. Waist circumference (WC) was measured at the level of the umbilicus to the nearest 0.1 cm with the use of a flexible fiberglass tape while the subjects were standing and without clothes [18]. The WC percentile was established following the WHO guidelines. All measurements were performed by three trained registered dietitians to control interobserver variability and data were the results of an average of the three measurements. Blood pressure was assessed using a pediatric baumanometer; three determinations were completed for each child, and the median was obtained.

### 2.3. Biochemical Measurements

Blood samples were collected from all participants after a 12 h overnight fast and kept at 2°C to 8°C for further centrifugation within the first three hours. Serum/plasma samples were stored at −80°C until measurements were performed. TG, glucose, and HDL-c were determined in serum with the use of clinical-grade reagents from Pointe Scientific (Canton, MI), following the manufacturer's instructions.

### 2.4. Plasma Cytokines and Irisin Analysis

As previously reported [19], cytokines analysis was performed using the LEGENDplex Human Inflammation panel (IL-1*β*, IFN-*α*, IFN-*γ*, TNF-*α*, MCP-1, IL-6, IL-8, IL-10, IL-12p70, IL-17A, IL-18, IL-23, and IL-33) for a multianalyte cytometric assay (BioLegend, San Diego, CA, USA). Each experiment was performed in triplicate with undiluted samples. Data were collected on the flow cytometer FACS-Canto II (Becton Dickinson, USA), and plasma cytokine concentration was calculated using the standard curve provided with the LEGENDplex software (BioLegend). Irisin plasma concentration was measured using ELISA kits (Avisera Bioscience Inc., SK00170-09, California, USA), following the manufacturer's instructions.

### 2.5. Identification Quantification of Plasma Fatty Acid Profiles (Phospholipid and Cholesteryl Esters Fatty Acids)

Lipids were extracted from plasma samples (100 *μ*L) by the Folch method using chloroform-methanol (2 : 1 *v*/*v*) [20]. Each lipid extract was fractionated using a solid-phase extraction aminopropyl column (500 mg, 3 mL, Bond Elut NH_2_, Agilent Technologies Inc., CA, USA) into five lipid fractions by the procedure described by Agren et al. [21], with slight modifications in the chromatographic determinations. The lipid fractions included cholesteryl esters (CE), TG, mono and diglycerides (MG+DG), free fatty acids, and phospholipid esters (PL) [21]. Cholesteryl nonadecanoate (400 ppm), from Nu Chek Prep Inc. (MA, USA), was added as an internal extraction standard. Eluted fractions were stored at -80°C until further quantification of FA compositions.

Prior to chromatographic analyses, the CE and PL fractions were independently evaporated to dryness in a vacuum concentrator (Centrivap, Labconco, MO, USA) and resuspended in 0.5 mL toluene-hexane 1 : 1 mixture. Subsequently, the FA in the plasma CE and PL fractions were transmethylated in tightly capped tubes by using methanol-H_2_SO_4_ 93 : 7 (1 mL, 93 : 7 **v**/**v**) and placed in a water-bath (80°C for 60 min). After cooling, hexane was added (3 mL), and the tubes were shaken (1 min).

Chromatographic peaks were identified by gas chromatography coupled to a mass spectroscopy detector (GC-MS, 7890B/5977A, Agilent Technologies Inc., CA, USA) using a standard external mixture of 39 fatty acid methyl esters (FAMEs; GLC 566, Nu Chek Prep Inc., MA, USA). The analytical column was fused-silica SP-2380 capillary column (100 m × 0.25 mm i.d., 0.2 *μ*m film thickness; Supelco, PA, USA). The oven temperature ramp ranges from 140°C to 240°C to 240'C at 4°C/min with a holding time of 45 min. The injector temperature was 260°C. Helium was used as a carrier gas at a flow rate of 1 mL/min. MS was managed in the electron impact mode at 70 eV with a scan range of 30–550 amu. The ion source and mass quadrupole temperatures were 150°C and 250°C, respectively. Individual FAs in plasma, CE, and PL were identified by comparing peak retention times with those of a standard external mixture and by comparing the peak's mass spectra with those of the NIST library (2017).

FAs were quantified as FAMEs by means of an Agilent 6850A gas chromatograph coupled with a flame ionization detector (GC-FID, Agilent Technologies Inc., CA, USA) using the same chromatographic parameters described for GC-MS identification. The flame ionization detector temperature was 300°C. The FA quantification of each peak, in parts per million (mg/kg), was obtained by introducing individual peak areas [pA · s] into the equation obtained from the calibration curve of cholesteryl undecanoate, which was used as an internal standard. The response factors for the quantification of each FA were calculated as indicated in the AOAC method 996.06 [22]. Cholesteryl undecanoate (600 ppm), obtained from Nu Chek Prep Inc. (Elysian, MA, USA), was spiked into each sample as an internal standard for quantification. The absolute concentrations (ppm) of individual FAs in each fraction (CE and PL) were normalized as relative percent weight concentrations of the total amount of FAs of each patient, as described by Agren (1992) [21].

Desaturase activities in plasma CE and PL were estimated by relating the amount of specific substrates to their corresponding products [23]. Ratios of FA bound, as CE, were determined as follows: stearoyl-CoA-desaturase (SCD) = (16 : 1n − 7/16 : 0), *Δ*6-desaturase (D6D) = (18 : 3n − 6/18 : 2n − 6), *Δ*5-desaturase (D5D) = (20 : 4n − 6/20 : 3n − 6). The ratios of FA in plasma PL were calculated similarly except for the D6D = (20 : 3n − 6/18 : 2n − 6) [24].

### 2.6. Statistical and Data Analysis

Hierarchical clustering analysis was performed using Euclidean distance and Ward agglomeration. Pearson correlation was used for the correlation analysis. Only complete data were used. Predictor trees were build using a rpart package in R (https://cran.r-project.org/). For systematic analyses of all variables, Mann-Whitney or Kruskal-Wallis test was performed for two or three groups, respectively. All of them, as well as principal component analyses, were performed in R.

Specific variables (CMR, PL, CE, irisin, and cytokine data) among the MHO, MUO, and NW groups were assessed using ANOVA with a Bonferroni correction for multiple comparisons to compare groups for parametric data and using the Kruskal-Wallis test with a Dunn's correction for multiple comparisons for nonparametric data. For two-group comparisons, unpaired Student's **t**-test for normal data or Mann-Whitney tests for nonnormal data were used. For normality, the D'Agostino-Pearson test was performed and declared nonnormal if a **p** < 0.05 was obtained in any of the groups. These analyses were performed in Prism (GraphPad Software, CA, USA).

## 3. Results

A total of 114 children, 58 females and 56 males, aged 6-12 years, participated in the study.  Table 1 shows the anthropometric parameters for the NW and the OB group. According to the-BMI percentile (BMIp), 34 children belonged to the NW group, whereas 80 children belonged to the OB group.  Table 2 shows the biochemical and the other associated parameters for the OB group, subdivided into MHO and MUO. MUO was found in 55% of the 80 children with obesity, and all presented high levels of TG (mean: 189 mg/dL) and low levels of HDL-c (mean: 33 mg/dL). Glucose concentrations were elevated in 9% of the children, and 31.8% showed an elevated blood pressure (systolic, diastolic, or both). In the MHO group, 36% of the children presented low HDL-c levels and 22% high TG levels. While age, the waist circumference percentile (WCp), and the BMIp did not differ between the groups, TG and diastolic blood pressure (dBP) were significantly higher, and HDL-c levels were significantly lower in the MUO group compared with the MHO group. There was no difference in glucose or systolic BP between the groups (Table 2).

**Figure 1:**
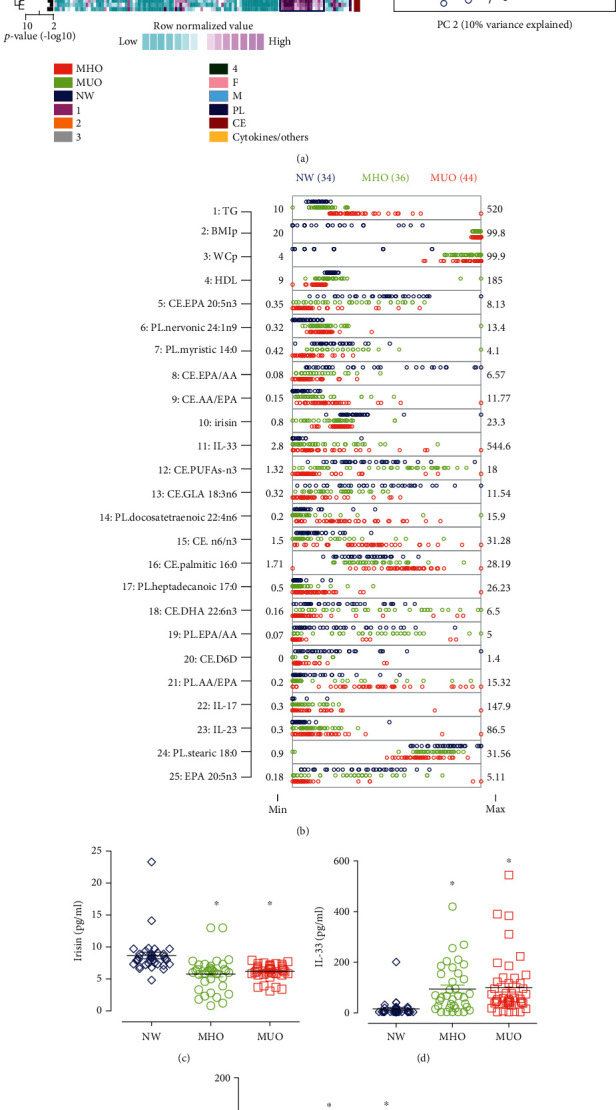
Integrated analysis across groups. (a) Profile of PL- and CE-FAs, cytokines and irisin concentrations, and anthropometric data. A principal component analysis confirms the overall clusters shown in the heat map showing the clustering of the children. With NW (blue), MHO (green), and MUO (red). The squares in the heat map enlighten the cluster of factors increasing in each group. (b) Top 25 most significant variables. The range is shown. (c–g) Selected variables. Kruskal-Wallis/Dunn tests were performed. Statistical difference vs. NW is represented with ^∗^; vs. MHO with ^#^, and vs. MUO with ^&^. AA: arachidonic acid; CE: cholesteryl ester; CMR: cardiometabolic risk; D6D: delta-6-desaturase; DHA: docosahexaenoic acid; EPA: eicosapentaenoic acid; GLA: *ɣ*-linoleic acid; IL-: Interleukin-; MHO: metabolically healthy obesity; MUO: metabolically unhealthy obesity; NW: normal weight; BMIp: body mass index percentile; PC: principal component; PL: phospholipid; WCp: waist circumference percentile; TG: triglycerides.

The analysis of FAs profiles, irisin, and cytokines concentration (Figure 1) demonstrated that children with MUO differed from the children with MHO and from those with NW with no gender differences (Figure 1(a)).  Figure 1(b) shows the 25 factors that differed the most between the groups. The top non-FA plasma factor was irisin concentration, which is about 30% lower in both MHO and MUO with respect to the NW group (Figure 1(c)). Meanwhile, cytokines were significantly higher in children with OB, independently of their metabolic condition, compared with the NW group. This is exemplified by IL-33 (six-fold higher in both, MHO and MUO; *p* < 0.001 and *p* < 0.0001, respectively) and IL-17 (six-fold higher in MHO and 10-fold higher in MUO, *p* < 0.001) and shown in Figures 1(d) and 1(e), respectively. These data indicate that irisin and cytokines are not useful for differentiating CMR clustering. However, the difference between children with MHO and MUO can be explained by the FA profile, as was observed for CE-eicosapentaenoic acid (CE-EPA, 20 : 5n3). The CE-EPA relative concentration was 55% lower in children with MUO compared with that in children with MHO (*p* < 0.0001), while the lowest relative concentration (70%) was found with respect to NW children (*p* < 0.0001). The CE-EPA relative concentration in children with MHO was 34% (*p* < 0.0001) lower than that in children with NW (Figure 1(f)). However, some FAs like PL-nervonic acid (20 : 1n9) presented an equally lower relative concentration in both, MUO and MHO, compared with that found in the NW group (two-fold, *p* < 0.0001; Figure 1(g)).

### 3.1. Increased Proinflammatory Cytokines and Irisin Concentrations in Obesity Do Not Differentiate the MHO and MUO Groups

Table 3 shows irisin and cytokines concentrations for the three groups, children with NW, those with MHO, and those with MUO. Irisin and most of the cytokines, except for IL-1*β* and IFN-*α*, differed significantly in children with MHO and MUO, compared with children from the NW group. Noteworthy, there was no significant difference in irisin concentration nor in the inflammatory cytokines between the MHO and MUO groups.

### 3.2. FA Profile Clusters Differentiates Children with MUO from Those with MHO

**Figure 2:**
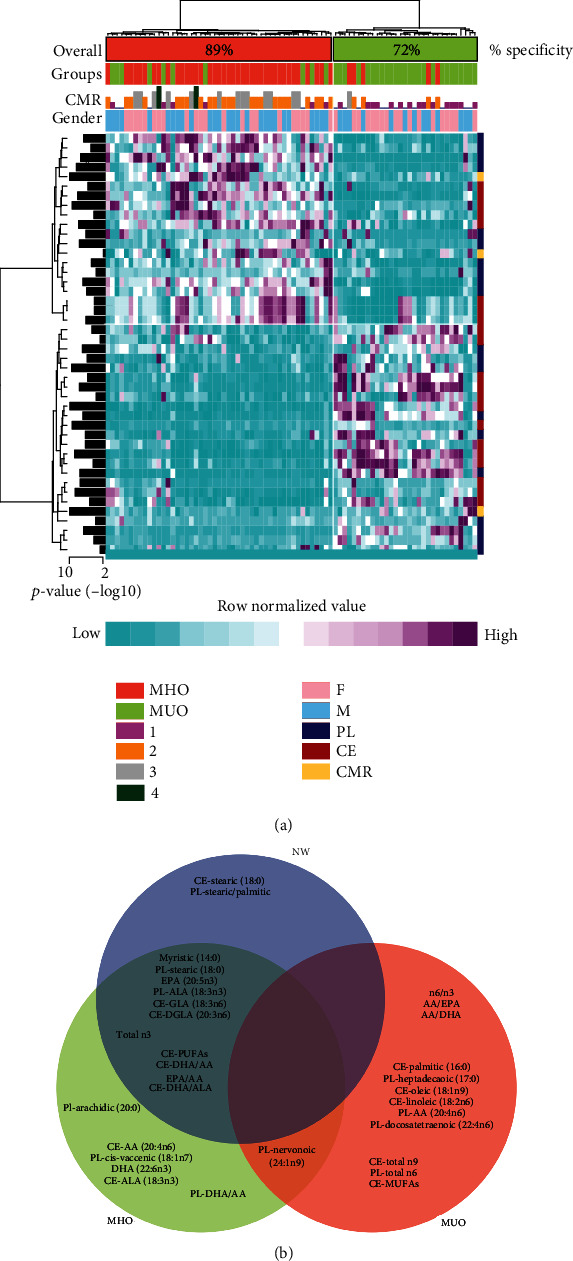
Increased fatty acids are associated with obesity and cardiometabolic risk. (a) Heat map of the variables that are significatively different between children with MHO (*n* = 36) and MUO (*n* = 44). (b) Venn diagram showing the clustering of statistically increased FAs in the different groups. Mann-Whitney test was performed. AA: arachidonic acid; ALA: *α*-linoleic acid; GLA: *ɣ*-linoleic acid; CE: cholesteryl ester; CMR: cardiometabolic risk factors; DGLA: dihomo-*ɣ*-linoleic acid; DHA: docosahexaenoic acid; EPA: eicosapentaenoic acid; MHO: metabolically healthy obesity; MUO: metabolically unhealthy obesity; NW: normal weight; PL: phospholipid.

To confirm that only the association of the FA profile explains the differences of CMR clustering (MHO vs. MUO), we subanalyzed only children with OB (Figure 2). We found clear specific FA clusters for ~72% of the children with MHO and ~89% of the children with MUO (Figure 2(a)). Remarkably, when we analyzed the cluster of FAs most represented in children with MHO and compared them with those FAs elevated in children with NW (*p* ≤ 0.01; Figure 1(a), blue square), we confirmed that the majority of the FAs that were higher in the MHO group were equally represented in the children with NW (88% concurring variables). This cluster of FAs were primarily associated with n-3 PUFAs, as well as myristic, stearic, and arachidic SFAs (Figure 2(b)). In the NW group, stearic acid (18 : 0) was the main FA present in both the PL and CE fractions. Furthermore, the stearic to palmitic ratio in both PL and CE fractions was significantly higher in children with NW and MHO than in those with MUO (*p* < 0.0001). On the other hand, children with MUO presented a significantly higher relative concentration of n-6 PUFAs. FAs relative concentrations in all groups are shown in Supplementary Tables 1 and 2 and the distribution of PL and CE FAs profile in Supplementary Figure 1.

### 3.3. FA Profile Correlates with CRM in Children with MUO

**Figure 3:**
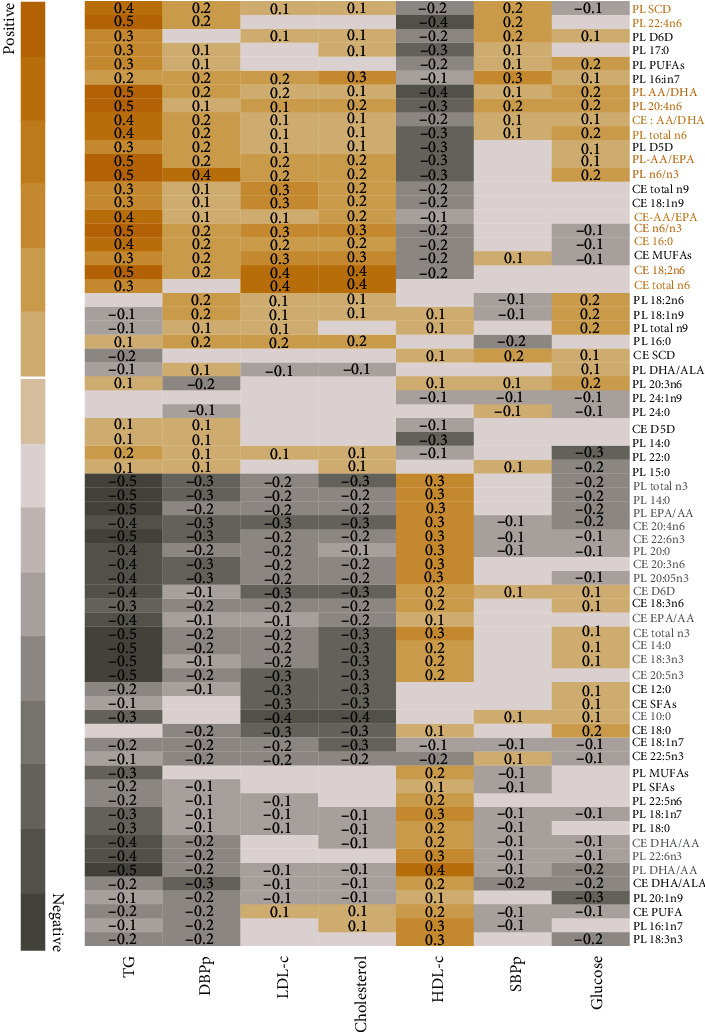
Correlation analysis between fatty acids and cardiometabolic risk factors in children. Only significant correlations are shown as inside cell values. DBPp: diastolic blood pressure percentile; HDL-c: high-density lipoprotein cholesterol; LDL-c: low-density lipoprotein cholesterol; SBPp: systolic blood pressure percentile; TG: triglycerides.

Furthermore, a correlation analysis (Figure 3) showed that all the FA with the highest concentrations present in children with MUO highly correlated (*r* = 0.3 to 0.5) with TG, whereas the PL-docosatetraenoic (22 : 4n6), PL- arachidic acid (20 : 4n6), and n-6/n-3 ratios (arachidonic acid (AA)/DHA, AA/EPA) correlated negatively with HDL-c (*r* = −0.4 or −0.3). This reinforced the finding that all children with MUO had TG concentrations above 110 mg/dL. On the contrary, the FAs that showed the highest concentrations in children with MHO, such as total n-3 FAs and myristic acid (14 : 0), showed a negative correlation with TG (*r* = −0.5 or −0.4) and cholesterol (*r* = −0.3 or −0.2) and a positive correlation with HDL-c (*r* = 0.3).

**Figure 4:**
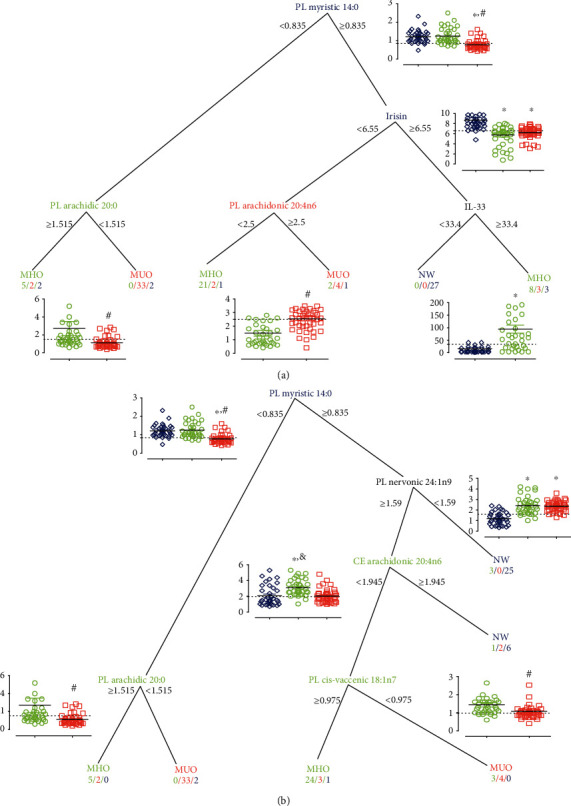
Decision tree analysis relating clinical groups (NW, MHO, and MUO). (a) Considering fatty acids, irisin, and cytokines concentration or (b) only fatty acids. The variable name, frequency, and cut-off values are shown. Inset plots showed the sample distribution of the corresponding variable and were cropped to highlight the media in each group. The numbers bellow group shows the “*n*”—number of children for MHO/MUO/NW. Statistical difference vs. NW are represented with ^∗^; with respect to MHO as ^#^, and with MUO as ^&^. CE: cholesteryl ester; MHO: metabolically healthy obesity; MUO: metabolically unhealthy obesity; NW: normal weight; PL: phospholipid.

To determine which FAs and other variables could be considered predictors of CMR risk, we performed a conditional inference three analysis (Figure 4) including all the variables (Figure 4(a)), as well as another analysis taking into account only the FAs (Figure 4(b)). In both cases, PL-arachidic acid (20 : 0) plasma relative concentrations (higher than 1.5 %ppm) distinguished MHO from MUO, particularly in children with a low proportion of myristic acid (14 : 0; 0.853 %ppm). Indicating that these FAs are surrogates of protection in children with obesity, whereas a higher proportion of PL-AA (20 : 4n6; 2.5 %ppm) is highly associated with CMR and, therefore, with a higher risk of developing CVD in children.

## 4. Discussion

One of the major concerns related to the incidence of obesity in childhood is the predisposition to develop CVD at this young age [1]. In response to this, data addressing the relation of dietary fat intake or the role of subtle inflammation in health youths with the risk of developing chronic diseases, such as T2D and CVD have emerged [24–26]. FA profiles have been described comparing children with OB and with NW [23]. However, only a few studies differentiate the FA profile in population with OB versus the population with OB that has already developed a CMR [27]. In the current study, we demonstrated that plasma cytokines and irisin concentrations are associated with OB but not with CMR. These data indicated that both irisin and cytokines are not suitable markers for differentiating CMR clustering, but rather, as previously described, they are mostly associated with obesity and thus they represent an independent factor for CVD. By contrast, we found FA profiles, specifically gathered by clusters in both, PL and CE fractions that differentiate MUO from MHO. n-3 PUFAs relative concentrations were the major FAs observed in the MHO and n-6 PUFAs in the MUO group, indicating that possibly, n-3 PUFAs might protect children from developing CMR.

In the northern México, from where our studied population belongs, fat intake among children is 10% above the national average. A higher prevalence of obesity among children aged 9–11 years has been described [28]. Among this population, the mean intake of n-6 PUFAs (mainly linoleic acid [LA], 97.4%) and palmitic acid is higher than that of their counterparts, n-3 PUFAs (predominantly *α*-linolenic acid [ALA], 87%), and stearic and myristic acids [29]. As FA composition is regulated, particularly in plasma PL and CE fractions, and each fraction reflects a unique FA pattern of dietary intake [27, 30], we evaluated both FA profiles. We found that MUO was associated with higher n-6 and n-9 FAs, whereas MHO was linked to higher n-3 FAs relative concentration. Even though we did not assess dietary intake, in the context of the findings of the studies described, our results might imply that the dietary intake of foods containing saturated fat is greater in the children with MUO compared to those with MHO, while children with MHO might include a higher intake of polyunsaturated fat in their diet.

We found that the concentration of EPA (20 : 5n3), DHA (22 : 6n3), CE-*ɣ*-linolenic acid (GLA; 18 : 3n6), and CE-dihomo-GLA (DGLA; 20 : 3n6) was higher in children with MHO, who also had lower TG concentrations and dBP level, but higher HDL-c concentrations, than children with MUO. Furthermore, we found a significant negative correlation between EPA, DHA, GLA, and CE- DGLA with TG and dBP, but positive with HDL-c. In line with these findings, other authors have reported that the intake of EPA (20 : 5 n3), DHA (22 : 6 n3), and GLA(18 : 3n6) increased the plasma concentration of PL-DGLA (20 : 3n6) and lowered TG concentrations along with beneficial CV outcomes [31]. Our results might indicate that these FAs are protective for the development of CMR factors in children with obesity. On the contrary, PL-AA (20 : 4n6), CE-LA (18 : 2n6), and PL-docosatetraenoic acid (22 : 4n6) were significantly higher in the MUO group, as were TG levels and d BP with a positive correlation with both variables, indicating a possible contribution of these FAs in the development of CMR factors which further increases the risk of developing CVD in children. This could be explained by the fact that their precursors, CE-oleic acid (OA; 18 : 1n9) and CE-LA (18 : 2n6) concentrations, were higher in MUO compared with that of PL-ALA (18 : 3n3), which was increased in MHO. Furthermore, a higher proportion of n-3 PUFAs (EPA; 20 : 5 n3, DHA; 22 : 6 n3, and ALA; 18 : 3n3) and a lower proportion of n-6 PUFAs (LA; 18 : n6 and AA; 20 : 4n6) found in the MHO group accounted for the lower the n-6 : n-3 ratio, compared with the MUO group. A higher n-6 : n-3 ratio has proved to be beneficial for CV health [32, 33], whereas the absence of n3-PUFAs in the diet favors LA (18 : 2n6) to increase the concentration of n-6 PUFAs and the n-6 : n-3 ratio, both related to the pathophysiology of CVD [34]. Our results show a high correlation of the total n-6 : n-3 ratio and of the AA/EPA and AA/DHA ratios with MUO, all of which have also been associated with CVD [35]. The data suggest that these ratios of FAs, in addition to the aforementioned individual FAs, are also key factors that either contribute to the development of or the prevention of CVD. Mechanistically, the effects of n-3 FAs are mediated through anti-inflammatory pathways and by favoring membrane fluidity that is associated with better signaling pathways, reflecting improvements in insulin sensitivity and immune cell function [36]. In this context, CE-GLA (18 : 3n6) was higher in MHO. This FA has been positively associated with insulin sensitivity in another study [37]. Additionally, GLA is metabolized to DGLA and incorporates phospholipids into the cell membrane; it competes with AA for cyclooxygenases and lipoxygenases, and it produces anti-inflammatory eicosanoids [38]. In the same way, n-3 PUFAs compete with such enzymes to form less active prostaglandins, (i.e., leukotriene B_5_ versus B_4_) [39]. In addition, n-3 PUFA dietary intake increases cholesterol efflux with respect to a diet rich in SFA [40] and avoids insulin resistance though specific receptors such as TLR4 and GPR120 [7, 41]. Another anti-inflammatory effect is through the secretion of lipid-derived mediators that use AA (20 : 4n6), EPA (20 : 5n3), and DHA(22 : 6n3) as precursors such as lipoxin and resolvins [42].

On the other hand, it is well established that SFAs are unhealthy [43]. Nevertheless, we found that myristic acid (14 : 0), stearic acid (18 : 0), and arachidic acid (20 : 0) are positively associated with MHO, whereas only palmitic acid (16 : 0) and heptadecanoic acid (17 : 0) are associated with MUO. This could be explained by the diet, based on the finding by Montoya et al. [40], in a controlled study comparing two different diets in healthy adults with NW. In those receiving a diet rich in n-6 and n-3 PUFAs (sunflower oil with and without fish oil, respectively), the concentration of PL-stearic acid increased compared with a diet rich in SFA (palm oil), in which palmitic acid augments, probably by altering the cholesterol efflux by the cells [40]. It has also been observed that moderate consumption of myristic acid improves plasma TG, HDL-c, and CE-DHA levels [44]. In line with these findings, our results show a negative correlation between myristic acid (14 : 0) and PL-stearic acid (18 : 0) relative concentrations with cholesterol, TG, and dBP but positive with HDL-c; opposite results were found for palmitic acid (16 : 0). In addition, it has been suggested that the ratio stearic to palmitic is more important than their individual value and that this ratio is a predictor of diabetes remission in obesity [45]. In this regard, we found that the stearic to palmitic acid ratio (PL fraction) is higher in children with NW and with MHO compared with MUO, which is in agreement with the previous study regarding a better prognosis. With respect to arachidic acid (20 : 0), we found it in higher concentrations in MHO compared with MUO. In agreement, a recent study demonstrated, after a follow-up of six years in of 4,249 participants, that higher concentrations of very long SFAs such as arachidic acid (20 : 0) are associated with a lower risk of heart failure with a hazard ratio of 0.72 (95% confidence interval, 0.59–0.88) [46]. Nevertheless, contrary to our findings related to the PL-heptadecanoic acid (17 : 0) plasma concentration—that was higher in children with MUO and correlated positively with TG, cholesterol, and BP but negatively with HDL-c—this FA has been inversely associated with CVD in older adults [47]. Finally, we reported higher concentrations of PL-cis-vaccenic acid (18 : 1n7) in children with MHO. Although the mechanism is not well studied, it has been proposed as a stronger predictor of insulin sensitivity and a lower risk of diabetes [37, 48].

Regarding inflammatory markers, our results indicate that the presence of CMR in our population is only associated with the FAs profile, suggesting that FAs might play a relevant role in its appearance. Although most of the described mechanisms modulate inflammation, we did not observe differences in the concentrations of cytokines or irisin between MUO and MHO, which suggests that CMR factors and inflammation are independent risk factors for developing CVD. We hypothesize that low-grade systemic inflammation mediated by cytokines mainly depends on adipose tissue hypertrophy and hyperplasia without influencing CMR factors, then acting as an independent risk factor. Meanwhile, FAs directly modulates immune cell activation promoting MUO. In this context, insulin resistance (present in most populations with obesity) depends on NLRP3 (NOD-, LRR-, and pyrin domain-containing protein 3) inflammasome activation affecting B and T cells response [49–51]. In this regard, MUFAs and PUFAs hamper NLRP3 activation, whereas SFAs act opposite [49]. Thus, our results might be explained by these set of mechanisms. It is worth mentioning that meanwhile MUO had an increased risk of developed CVD [17], our finding regarding inflammation indicates that children with MHO are still at risk since they do not differ in terms of inflammation from children with MUO, they are both equally increased relative to the NW group. Supporting findings showed that children with obesity in the absence of CMR factors showed left ventricular hypertrophy associated with BMI [52]. Moreover, it was recently demonstrated that chronic inflammation predicts all-cause mortality, including CVD [53, 54].

Other works also support that the association among cytokines in obesity in children is independent of CMR [55–57]. Sontichai et al. showed that Thai children with obesity aged 6 to 8 years had elevated TNF-*α*, IL-6, and C-reactive protein (CRP) with no increase concerning CMR [26]. Furthermore, a study in 446 children (aged 6–12 years) found that TNF-*α*, IL-6, and IL-8 increased in children with obesity compared to the NW group and that inflammation and anthropometric and metabolic features are independent risk factors for CVD [57]. Moreover, in adults (aged 45–84 years), in a population study of 6,814 participants with a median follow-up time of four years, found that inflammatory markers (IL-6 and CRP) are independent predictors of heart failure from obesity [58]. Although the irisin role is controversial [9, 14], increased irisin concentration inhibits the secretion of proinflammatory cytokines [9]. In this regard, we found a negative correlation (-0.2 to -0.3) of irisin with IL-23, IL-33, TNF-*α*, IL-17, IL-12 e IL-8 (data not shown) reinforcing the “irisin-anti-inflammatory axis” that we previously proposed [13].

Despite, we previously reported that irisin plasma concentrations increased in children about obesity (10 children per group) with a positive correlation to the BMI percentile [14], and other reports showed an association between higher levels of irisin and an unhealthier FA profile [59]. In the present study, in a larger population, we observed that decreased irisin concentrations were associated with obesity but not with the FA profile since lower levels of irisin were associated with obesity even in the children with MUO that showed the worst FA profile. In line with this work, a recent study in a cohort of 96 children aged 6–10 years also found that lower concentrations of irisin in plasma were associated with obesity, with an inverse correlation with TG and glucose [60]. In the present work, we also found a negative relationship with TG (*r* = −0.3) and positive (*r* = 0.2) with HDL-c but none with glucose concentrations (data not shown). In accordance, we recently found that irisin correlates negatively with obesity and TG in children (7-17 years old) with T2D [13].

However, this study has some limitations. Since it was not a longitudinal study and was conducted in a population of one ethnicity (Hispanic) from one region in the north of Mexico, causality, generalizability, and extrapolation of the results cannot be established. Dietary intake and physical activity were not assessed. Nevertheless, the study has some strengths. We purposely conducted the study in children with obesity with the same BMIp and WC, same age span, and equal gender distribution, which enabled an analysis of the studied variables without these confounders. Our analysis is the first of its kind and was difficult to set a sample size *a priori*. Nevertheless, with our current design and data, we were able to establish statistical differences among groups suggesting that samples used were sufficient for a preliminary characterization and that are representative of major groups.

## 5. Conclusions

Defining CVR based on CMR factors allows establishing an MUO in those children with two or more CMR. We found that FA profiles of the PL and CE fractions differentiate children with MHO from MUO independent of age, sex, BMI, and WCp. On the other hand, chronic systemic inflammation (irisin decreased levels and elevated inflammatory cytokines) remains equally in both groups with obesity differing from children within the NW group, indicating that inflammation is an independent risk factor to developed CVD. Given these results and considering that n-3 and n-6 are directly associated with dietary intake of fats, it would be advisable to follow the recommendations provided by national associations to increase PUFAs intake over SFAs to reduce CMR and consequently CVR. An adequate proportion of the n-3 : n-6 FA might induce conversion of the MUO to the MHO phenotype and, consequently, to the reduction in the risk of developing T2D and CVD. However, counteracting obesity is also essential to avoid inflammatory CVR. Future studies regarding the clustering of FAs as predictive of CMR in the pediatric population are needed in order to provide particular dietary recommendations.

## Figures and Tables

**Table 1:** Clinical characteristics of the study population (*n* = 114).

Variables	NW *n* = 34	OB *n* = 80	*p* value
Males/females (*n*)	16/18	40/40	
Age (years)	9 (7-10) (6–12)	9 (7.25-10) (6–12)	0.6089
BMI (percentile)	30 (24.25-46.25)	99 (97.63-99)	<0.0001
WC (percentile)	25 (13.75-50)	94.25 (89.8-98.7)	<0.0001

Data is presented as median and interquartile range. Tests performed with Mann-Whitney as described in Methods. NW: normal weight; OB: obesity; BMI: body mass index; WC: waist circumference.

**Table 2:** Cardiometabolic risk factors of population with obesity (*n* = 80).

	MHO	MUO	*p* value
Males/females (*n*)	16/20	24/20	
Cardiometabolic risk factors			
Glucose (mg/dL)	81.5 (77.25-85.75)	85 (78.25-92)	0.1310
TG (mg/dL)	91 (75-108)	170 (132.8-207.3)	<0.0001
HDL-c (mg/dL)	41.5 (38-48.5)	33.5 (31-36)	<0.001
BP: systolic/ diastolic (percentile)	64.44 ± 18.25/ 40.92 ± 15.01	71.45 ± 22.26/ 52.23 ± 19.39	0.133/ 0.005
Associated factors			
Age (years)	8 (7.25-10)	9 (7.25-11)	0.2165
WC (percentile)	93.15 (87.4-97.8)	95.5 (91.05-99.3)	0.1349
BMI (percentile)	98.24 ± 1.07	98.52 ± 1.09	0.2557

Data is presented as median and interquartile range for nonparametric data and as mean ± s.d. for parametric data. BP: blood pressure; MHO: metabolically healthy obesity; MUO: metabolically unhealthy obesity; TG: triglycerides; HDL-c: high density lipoprotein cholesterol; WC: waist circumference; BMI: body mass index. Tests performed with ANOVA/Bonferroni or Kruskal-Wallis/Dunns, as described in Methods.

**Table 3:** Irisin and cytokines concentration (pg/mL) in children with NW, MHO, and MUO.

	NW (*n* = 34)	MHO (*n* = 36)	MUO (*n* = 44)	*p* value
Irisin	8.05 (7.3-8.95)	6.15 (4.1-6.9)^∗^	6.4 (5.8-7)^∗^	<0.0001
IL-1*β*	0.3 (ND-0.3)	0.3 (0.3-0.3)	0.3 (0.3-0.55)	0.5897
IFN-*α*	6.15 (2.8-12.8)	5 (1.3-49.35)	5 (2.4-22.4)	0.8974
IFN-*ɣ*	11.5 (0.4-17.7)	16.85 (6.4-29.7)	18.1 (6.5-28.15)	0.0456
TNF-*α*	0.7 (0.4-0.7)	4.3 (0.7-14)^∗^	2.1 (0.7-13.28)^∗^	0.0003
MCP-1	152.8 (115.3-217.7)	188.1 (143.9-242)	220.3 (154-255.5)^∗^	0.0318
IL-6	1 (0.5-3.7)	3.5 (0.5-9)^∗^	2.4 (1.1-5.6)	0.0278
IL-8	1.3 (0.6-2.6)	3.1 (1.5-8.6)^∗^	3.9 (1.1-6.7)^∗^	0.0032
IL-10	1.35 (0.6-2.7)	3.15 (1.3-4.5)^∗^	3.1 (0.6-5.9)^∗^	0.0093
IL-12	0.9 (0.7-1.9)	2.65 (1.2-3.5)^∗^	2.3 (0.8-3.5)^∗^	0.0013
IL-17A	0.3 (0.3-0.3)	5.45 (0.35-15.23)^∗^	4.25 (0.3-22.4)^∗^	<0.0001
IL-18	84.45 (50.25-167)	121.8 (81.8-212.5)	145.1 (110.7-199.8)^∗^	0.0124
IL-23	2.85 (0.3-4.8)	8.6 (4.85-14.3)^∗^	8.3 (5.1-16.4)^∗^	<0.0001
IL-33	3.6 (3.6-18.5)	63.95 (20.8-155.2)^∗^	54.65 (38.7-121.1)^∗^	<0.0001

Data is presented as median and interquartile range for nonparametric data and as mean ± s.d. for parametric data. Tests performed with ANOVA/Bonferroni or Kruskal-Wallis/Dunn as described in Methods. Statistical difference vs. NW is represented with ^∗^. IL: interleukin; TNF: tumor necrosis factor; IFN: interferon; MCP: monocyte chemoattractant protein; MHO: metabolically healthy obesity; MUO: metabolically unhealthy obesity; NW: normal weight.

## Data Availability

The data used to support the findings of this study are available from the corresponding author upon request.

## Supplementary Materials

Supplementary Materials Supplementary Table 1: PL-Plasma FA relative concentration (%ppm) in children with NW, MHO, and MUO. Supplementary Table 2: CE-Plasma FA relative concentration (%ppm) in children with NW, MHO, and MUO. Supplementary Figure 1: PL and CE FAs profile.
