# Supplementary material for: Integrative Analysis of Lipid Profiles in Plasma Allows Cardiometabolic Risk Factor Clustering in Children with Metabolically Unhealthy Obesity
Source: Oxid Med Cell Longev. 2020 Aug 11;2020:2935278. doi: 10.1155/2020/2935278 (PMC7710435; doi:10.1155/2020/2935278)
Supplement: Supplementary Materials — Supplementary Table 1: PL-Plasma FA relative concentration (%ppm) in children with NW, MHO, and MUO. Supplementary Table 2: CE-Plasma FA relative concentration (%ppm) in children with NW, MHO, and MUO. Supplementary Figure 1: PL and CE FAs profile. [file 2935278.f1.docx]

Integrative analysis of lipid profiles in plasma allows cardiometabolic risk factor clustering in children with metabolically unhealthy obesity.

Elena Cristina Castillo, PhD^1,&^, Leticia Elizondo-Montemayor, MD^1,2, &,*^, Carmen Hernández-Brenes, PhD^3,4^, Dariana G. Rodríguez-Sánchez, PhD^3,4^, Christian Silva-Platas, PhD^1^, Luis Martín Marín-Obispo, MSc^3,4^, Nora A. Rodríguez- Gutierrez, MD^5^, Víctor Treviño, PhD^6^ and Gerardo García-Rivas, PhD^1,7*^.

| **Supplementary Table 1**. PL-Plasma FA relative concentration (%ppm) in children with NW, MHO and MUO. | | | | | |
| --- | --- | --- | --- | --- | --- |
|  |  | NW | MHO | MUO | *p-value* |
| SFAs | **SFAs sum** | 65.8 (62.5-68.1) | 63.6 (62.1-67.3) | 62.7 (59.5-66.4)^*^ | **0.0423** |
|  | Lauric, 12:0 | 0.5 (0.4-0.6) | 0.55 (0.3-0.9) | 0.7 (0.6-0.9)^*^ | **0.0131** |
|  | Myristic, 14:0 | 1.2 (1-1.4) | 1.1 (1-1.6)^&^ | 0.7 (0.6-0.8)^*,#^ | **<0.0001** |
|  | Pentadecanoic, 15:0 | 0.8 (0.55-1.4) | 0.8 (0.6-1) | 0.9 (0.6-1.55) | 0.2249 |
|  | Palmitic, 16:0 | 31.5 (29.6-33) | 31.3 (28.7-34) | 31.4 (29.28-33.6) | 0.9738 |
|  | Heptadecanoic, 17:0 | 1 (0.8-1.2) | 1.2 (0.9-1.75)^&^ | 3.1 (1.7-4.8)^*,#^ | **<0.0001** |
|  | Stearic, 18:0 | 26.6 (24.3-28.3) | 24.6 (21.9-26.1)^*,&^ | 21.37 (20-29.4) ^*,#^ | **<0.0001** |
|  | Arachidic, 20:0 | 1.4 (0.8-1.8) | 1.5 (1.1-2.3)^&^ | 0.9 (0.7-2.8) ^*,#^ | **0.0002** |
|  | Behenic, 22:0 | 1.4 (1-1.7) | 1.6 (1.3-1.8) | 1.7 (1.5-2)^*^ | **0.0072** |
|  | Lignoceric, 24:0 | 1.6 (1.2-2.1) | 1.3 (1-1.9) | 1.4 (1.1-1.6) | 0.0616 |
| MUFAs | **MUFAs sum** | 12.1 (10.9-13.4) | 13.1 (11.9-14.5) | 12.1 (11.3-13.1)^#^ | **0.0261** |
|  | **n9 sum** | 8.7 (7.5-9.6) | 8.35 (7-9) | 7.6 (7.1-8.7)^*^ | **0.0403** |
|  | Palmitoleic, 16:1n7 | 0.8 (0.7.1) | 1 (0.6-1.4) | 0.7 (0.5-2.7) | 0.0666 |
|  | Oleic, 18:1n9 | 7.4 (6-8.35) | 7.65(6.4-8.4) | 7.15 (6.2-8.1) | 0.06702 |
|  | Cis-vaccenic, 18:1n7 | 1.1 (0.95-1.3) | 1.3 (1-1.6)^&^ | 1 (0.8-1.1)^#^ | **0.0015** |
|  | Eicosadienoic, 20:1n9 | 1.2 (0.8-1.7) | 0.6 (0.4-0.9)^*^ | 0.4 (0.4-0.7)^*^ | **<0.0001** |
|  | Nervonic, 24:1n9 | 1.1 (0.6-1.6) | 2.25 (1.7-2.9)^*^ | 2.4 (2-2.7)^*^ | **<0.0001** |
| PUFAs | **PUFAs sum** | 22.27±4 | 22.7±3.1 | 24.4±4.1^#^ | **0.0336** |
|  | **n3 sum** | 4.7 (2.7-5.6) | 4 (3.1-5.75)^&^ | 2.2 (1.8-3.2)^*,#^ | **<0.0001** |
|  | **n6 sum** | 18.3 (15.9-20.4) | 18.65 (16.7-21.3)^&^ | 22.6 (20.2-25.8) ^*,#^ | **<0.0001** |
|  | Linoleic, 18:2n6 | 12.6 (9.9-14.2) | 12.3 (10.9-14) | 12.1 (10.45-13.9) | 0.8672 |
|  | ALA, 18:3n3 | 0.9 (0.6-1.2) | 0.6 (0.4-1.1)^&^ | 0.4 (0.3-3.95)^*,#^ | **<0.0001** |
|  | DGLA, 20:3n6 | 1.2 (0.6-1.7) | 1.6 (0.7-1.9)^*^ | 1.4 (1.2-1.9) | **0.0381** |
|  | AA, 20:4n6 | 1.8 (1.1-2.4) | 1 (0.7-1.9)^&^ | 2.6 (2-3.1) ^*,#^ | **<0.0001** |
|  | EPA, 20:5n3 | 1.9 (0.75-2.6) | 1.8 (0.52-2.2)^&^ | 0.4 (0.3-0.5) ^*,#^ | **<0.0001** |
|  | DTA, 22:4n6 | 0.9 (0.6-1.3) | 0.75 (0.4-1.5)^&^ | 4.2 (2.9-6.8) ^*,#^ | **<0.0001** |
|  | DPA (Osbond), 22:5n6 | 0.55 (0.4-0.7) | 0.7 (0.5-0.8) | 0.54 (0.5-0.7) | 0.1323 |
|  | DPA (Cupladonoic), 22:5n3 | 0.8 (0.5-2.3) | 0.9 (0.6-1.6) | 0.9 (0.7-1) | 0.4352 |
|  | DHA, 22:6n3 | 0.75 (0.4-1.2) | 0.7 (0.5-1.2)^&^ | 0.5 (0.4-0.6) ^*,#^ | **<0.0001** |
| Ratios | **n6/n3** | 3.9 (2.7-6.6) | 4.65 (3.1-6.5)^&^ | 10.2 (7.5-12.2)^*,#^ | **<0.0001** |
|  | AA/DHA | 1.8 (1.1-4.8) | 1.25 (0.6-4.25)^&^ | 5.1 (4.6-6.4)^*,#^ | **<0.0001** |
|  | AA/EPA | 0.8 (0.5-2.7) | 0.5 (0.4-4)^&^ | 6.7 (4.4-8.3)^*,#^ | **<0.0001** |
|  | DHA/AA | 0.5 (0.2-0.9) | 0.85 (0.2-1.6)^&^ | 0.2 (0.16-0.21)^*,#^ | **<0.0001** |
|  | EPA/AA | 1.2 (0.4-2) | 1.9 (0.2-2.9)^&^ | 0.15 (0.1-0.2)^*,#^ | **<0.0001** |
|  | DHA/ALA | 0.9 (0.5-1.3) | 1.15 (0.8-1.9)^*^ | 1.2 (0.8-1.5) | **0.0271** |
| Desaturases | SCD | 0.03 (0.02-0.03) | 0^*,&^ | 0.02 (0.02-0.03)^b^ | **<0.0001** |
|  | D6D | 0.15 (0.1-0.2) | 0.1 (0.1-0.2)^&^ | 0.2 (0.2-0.25)^*,#^ | **<0.0001** |
|  | D5D | 1.5 (0.85-2.3) | 0.6 (0.4-1.3)^*,&^ | 1.65 (1.3-2)^#^ |  |
| Data is presented as median and interquartile range for nonparametric data and as mean ± s.d. for parametric data. Tests performed with ANOVA/Bonferroni or Kruskal-Wallis/Dunn as shown in Methods. Statistical difference *vs* NW is represented with *; *vs* MHO with #, and *vs* MUO with &. AA: arachidonic acid; ALA: α-linolenic acid; D5D: Delta-5-desaturase; D6D: Delta-6-desaturase; DGLA: dihomo-ɣ-linolenic acid; DHA: docosahexaenoic acid; DPA: docosapentaenoic acid; DTA: docosatetraenoic acid; EPA: eicosapentaenoic acid; GLA: ɣ-linolenic acid; MHO: metabolically healthy obesity; MUO: metabolically unhealthy obesity; NW: normal weight; PL: phospholipid esters; SCD: Stearoyl-CoA-desaturase. | | | | | |

| **Supplementary Table 2**. CE-Plasma FA relative concentration (%ppm) in children with NW, MHO and MUO. | | | | | |
| --- | --- | --- | --- | --- | --- |
|  |  | NW | MHO | MUO | *p-value* |
| SFAs | **SFAs sum** | 39.6 (27.9-47) | 33.5 (28.2-39.6) | 34.1 (31.2-38.1) | 0.205 |
|  | Decanoic, 10:0 | 8.1 (3.1-10.6) | 7.05 (4.5-7.85) | 4.5 (3.3-6.6)^*^ | **0.0243** |
|  | Lauric, 12:0 | 7.2 (2.8-9.7) | 5.3 (3.6-6.7) | 4.2 (2.7-5.9)^*^ | **0.0351** |
|  | Myristic, 14:0 | 6 (3-8.3) | 4 (2.8-5.7)^&^ | 2.3 (1.7-3.2)^*,#^ | **<0.0001** |
|  | Palmitic, 16:0 | 12.25 (9.6-14.4) | 12.2 (10.45-15.1)^&^ | 17.7 (15.5-19.4)^*,#^ | **<0.0001** |
|  | Stearic, 18:0 | 8.2 (4.65-9.4) | 5 (3.8-6.1)^*^ | 4.6 (3.7-5.75)^*^ | **0.0027** |
| MUFAs | **MUFAs sum** | 18.8±5.7 | 21.8±5.5^*,&^ | 25.8±4.6^*,#^ | **<0.0001** |
|  | Palmitoleic, 16:1n7 | 2.2 (1.4-3.1) | 3.2 (2.4-3.75)^*^ | 3.7 (3.1-4.5)^*^ | **<0.0001** |
|  | Oleic, 18:1n9 | 14.8±4.8 | 15.6±4.9^&^ | 19.3±4.4^*,#^ | **<0.0001** |
|  | Vaccenic, 18:1n7 | 1.5 (1-2) | 2.6 (1.8-3.9)^*^ | 1.9 (1.5-2.6)^*^ | **<0.0001** |
| PUFAs | **PUFAs sum** | 41.6 (37-46.6) | 44 (40.8-47.9)^&^ | 39 (34.6-42.1)^#^ | **0.0014** |
|  | **n3 sum** | 7.1 (5.9-10.3) | 10.35 (4.2-13.7)^&^ | 2.6 (2.2-3.6)^*,#^ | **<0.0001** |
|  | **n6 sum** | 34.5±6.15 | 34.18± 5 | 35.29±5.4 | 0.6467 |
|  | Linoleic, 18:2n6 | 21.6 (16.6-21) | 24 (20.5-27.8)^&^ | 30.1 (25.6-33.6)^*,#^ | **0.0002** |
|  | ALA, 18:3n3 | 1.5 (1-2.7) | 3.65 (1.75-5.6)^*,&^ | 1.3 (1-1.9)^*^ | **<0.0001** |
|  | GLA, 18:3n6 | 4.9 (2.9-7.3) | 2.9 (1.5-3.8)^*,&^ | 1.2 (0.9-2.2)^*,#^ | **<0.0001** |
|  | DGLA, 20:3n6 | 4.8 (1.8-5.7) | 3.75 (2.4-5.2)^c^ | 1.6 (1.1-2.6)^*,#^ | **<0.0001** |
|  | AA, 20:4n6 | 1.5 (1-3.1) | 3 (2.3-3.9)^*,&^ | 1.85 (1.3-2.4)^#^ | **<0.0001** |
|  | EPA, 20:5n3 | 4.2 (2.9-5.4) | 2.7 (1.3-4.3)^*,&^ | 0.8 (0.6-1.1)^*,#^ | **<0.0001** |
|  | DHA, 22:6n3 | 1.3 (0.7-2.2) | 2.65 (1-4.6)^&^ | 0.5 (0.3-0.8)^*,#^ | **<0.0001** |
| Ratios | **n6/n3** | 4.7 (3.1-6.3) | 3.1 (2.5-8.5)^&^ | 13.6 (10.25-15.6)^*,#^ | **<0.0001** |
|  | AA/DHA | 1.3 (0.9-2) | 1.15 (0.8-2.6)^&^ | 3.8 (2.1-6.4)^*,#^ | **<0.0001** |
|  | AA/EPA | 0.5 (0.2-0.85) | 1.35 (0.8-2)^&^ | 2.1 (1.4-2.9)^*^ | **<0.0001** |
|  | DHA/AA | 0.8 (0.5-1.1) | 0.85 (0.4-2.1)^&^ | 0.3 (0.15-2.3)^*,#^ | **<0.0001** |
|  | EPA/AA | 2.2 (1.2-4.9) | 0.75 (0.5-1.3)^*,&^ | 0.5 (0.35-0.7)^*,#^ | **<0.0001** |
|  | DHA/ALA | 0.7 (0.55-0.9) | 0.6 (0.4-1.2)^&^ | 0.45 (0.25-0.6)^*,#^ | **0.0003** |
| Desaturases | SCD | 0.2 (0.1-0.25) | 0.3 (0.2-0.3)^8^ | 0.2 (0.2-0.3) | **0.0291** |
|  | D6D | 0.2 (0.1-0.4) | 0.1 (0.1-0.3)^&^ | 0.04 (0.03-0.07)^*,#^ | **<0.0001** |
|  | D5D | 0.5 (0.2-1.3) | 0.9 (0.6-1.4) | 1.25 (0.7-1.6)^*^ | **0.0041** |
| Data is presented as median and interquartile range for nonparametric data and as mean ± s.d. for parametric data. Tests performed with ANOVA/Bonferroni or Kruskal-Wallis/Dunn as shown in Methods. Statistical difference *vs* NW is represented with *; *vs* MHO with #, and *vs* MUO with &. AA: arachidonic acid; ALA: α-linolenic acid; CE: cholesteryl esters; D5D: Delta-5-desaturase. D6D: Delta-6-desaturase; DGLA: dihomo-ɣ-linolenic acid; DHA: docosahexaenoic acid; EPA: eicosapentaenoic acid; GLA: ɣ-linolenic acid; MHO: metabolically healthy obesity; MUO: metabolically unhealthy obesity; NW: normal weight; SCD: Stearoyl-CoA-desaturase. | | | | | |

|  |
| --- |


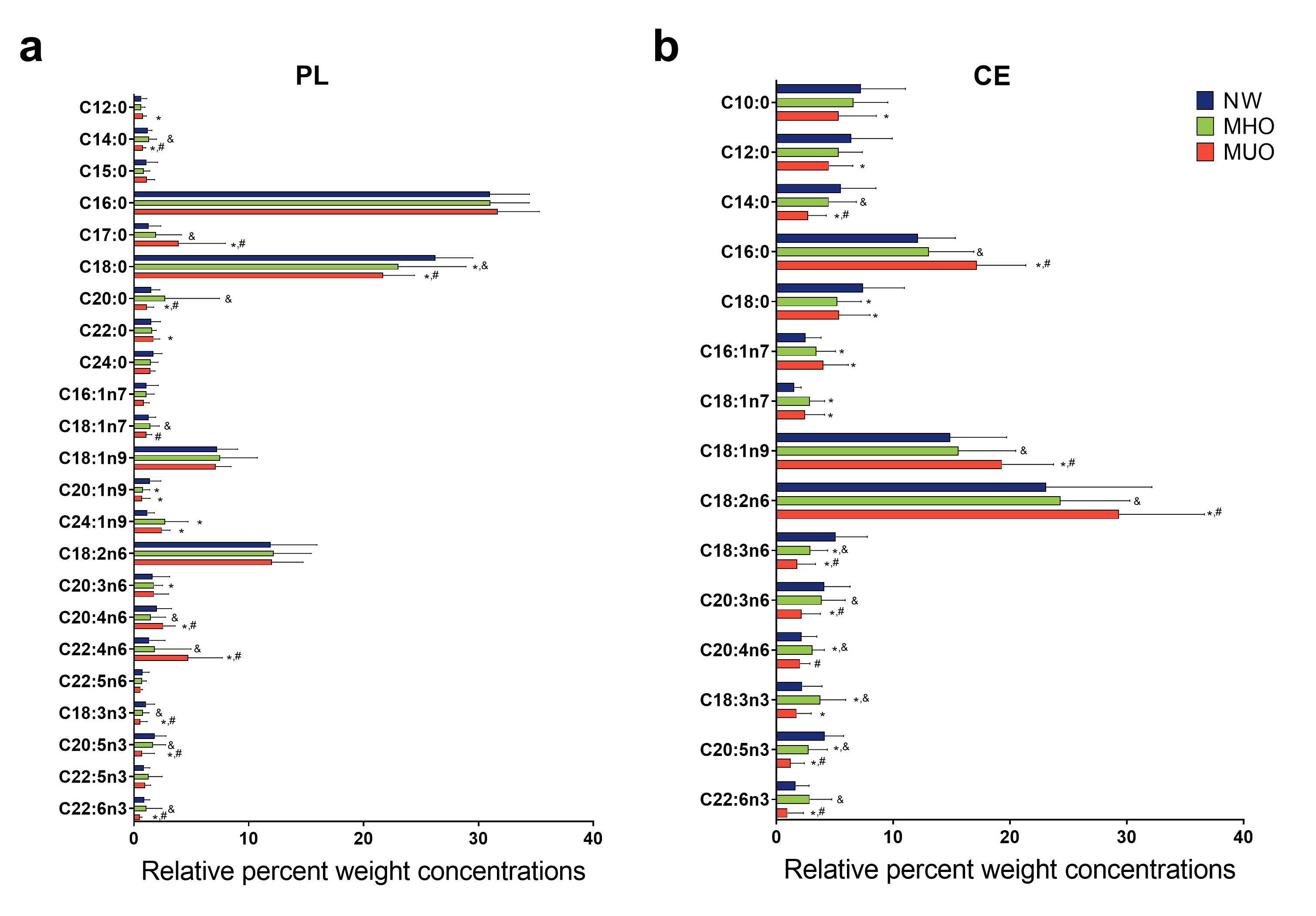


**Supplementary Figure 1.** PL and CE FAs profile. Data are presented as mean ± s.d. Tests performed with ANOVA/Bonferroni or Kruskal-Wallis/Dunns as shown in Methods. Statistical difference *vs* NW is represented with *; *vs* MHO with #, and *vs* MUO with &.
